# To the Editors of the Medical and Physical Journal

**Published:** 1802-12-01

**Authors:** 


### *To the Editors of the Medical and Phyfical Journal.*

GENTLEMEN,

IN common with many of your readers, I have felt myfelf much obliged to Mr. Ward for his valuable Communications on Opiate Frictions. I am inclined too to adopt his opinion of the fedative properties of opium: but cannot help thinking that in his inveftigation of its modus operandi, (a queftion in the higheft degree recondite, and perhaps of very little practical utility) he has been led into many hafty affertions, and has drawn fome unfounded conclufions. His cenfures of Dr. Crump's Treatife appear unwarrantably fevere, and needed not the additional aid of *italics* and *capitals* to render them more pointed.

In his laft letter, Mr. Ward has himfelf advanced an affertion, which appears, *prima facie*, moft glaringly falfe and untenable: namely, that alcohol is a fedative. Now, the purport of this letter is, to requeft that Gentleman to ftate the arguments which he confiders as corroborative of his opinion. But that we may not difpute about terms, I would likewife beg the favor of him to give his definition of the terms ftimulant and fedative: For if the former term be applied, as it moft commonly is, to certain fubftances which excite, for a time, the action of the heart and arteries, increafe mufcular power, and the fenforial functions, I do not perceive how he can poffibly exclude alcohol from the lift, unlefs, indeed, he confider the ftate of debility confequent on drunkennefs as a proof of its poffeffing a direct fedative power; if fo, his judgment is, I apprehend, widely different from that of other phyfiologifts.

I am, &c.

*Hatton Garden, Oct.* 10, 1803. C. C. W.

